# Expressional dynamics of minisatellite 33.15 tagged spermatozoal transcriptome in *Bubalus bubalis*

**DOI:** 10.1186/1471-2164-10-303

**Published:** 2009-07-07

**Authors:** Jyoti Srivastava, Sanjay Premi, Sudhir Kumar, Sher Ali

**Affiliations:** 1 Molecular Genetic Laboratory, National Institute of Immunology, Aruna Asaf Ali Marg, New Delhi – 110067, India; 2 Department of Zoology, University of Lucknow, Lucknow 226 006, UP, India

## Abstract

**Background:**

Transcriptionally quiescent spermatozoa have been established to be a repository of mRNA coding for several functionally essential cellular proteins. This entourage of mRNA is envisaged to be involved in post-fertilization and early embryogenesis. Minisatellites tagged with mRNA transcripts have been implicated with gene organization, regulation and function. However, the organization and expression of the minisatellite tagged transcript diversity, particularly in spermatozoa, remains unclear.

**Results:**

In the present study, we identified and characterized 12 mRNA transcripts from the spermatozoa of water buffalo *Bubalus bubalis *employing minisatellite associated sequence amplification (MASA) and a consensus sequence of 33.15 repeat loci. Of these 33.15 tagged transcripts, only one was found to be homologous to Bovine steroid 21-hydroxylase (P-450-c21) gene. Other ten transcripts showed significant similarity with various mRNAs or chromosomal contigs across the species. The remaining one construed to be novel since this was unreported in the database (NCBI GenBank). All these uncharacterized and known transcripts showed highest expression in testis and spermatozoa compared to that in somatic tissues and ovary. Of these 12 mRNA transcripts, 4 showed differential expression in the forebrain and hindbrain of buffalo. Moreover, genes corresponding to all the 33.15 tagged spermatozoal transcripts were found to be conserved across 13 other species analyzed.

**Conclusion:**

Our results show MASA as an important tool to capture mRNA transcript diversity tagged with minisatellites in the spermatozoa. Comprehensive characterization of these transcripts is envisaged to augment our understanding on the genes involved in testicular functions and sustenance of a viable paternal genome during pre- and post- fertilization events and early stages of development. Prospects of this approach in genome analysis in general and comparative genomics in particular are highlighted.

## Background

Ejaculated spermatozoa represent terminally differentiated cells in which transcription and/or translation of nuclear encoded mRNAs are considered to be unlikely. Therefore, the paternal genome is the only cargo carried by the spermatozoa to the ooplasm. Discovery of several transcription factors, soluble signaling molecules and structures delivered by spermatozoan into the zygotic cytoplasm upon fertilization have changed this perception [1-3]. Despite the transcriptionally inactive state, spermatozoa retain an entourage of mRNA transcripts encoding transcription factors and proteins involved in signal transduction, cell proliferation, chromatin condensation, regulation of sperm motility, capacitation and acrosome reaction [2-7]. Delivery of such spermatozoal transcripts to ooplasm is envisaged to have their potential significance during fertilization, embryogenesis and morphogenesis. Approximately 3000–5000 mRNA transcripts have been suggested to be present in the spermatozoa [2-7]. However, these remain to be characterized for their organization, expression and association with different regulatory elements and repetitive sequences.

Repetitive DNA sequences are dynamic components of the genome encompassing transposable elements, major satellites, minisatellites and microsatellites [8,9]. Most of these repeats are found in the non-coding regions of the genomes while a small fraction is retained within the transcriptome [10-12] and participate in gene regulation through transcription, translation or gene silencing [13-15]. Surprisingly, organization of the repeats within the transcriptomes of any mammalian species, particularly in the spermatozoa has remained undeciphered. To explore the organization of such repeats and the repeat tagged genes, we undertook the transcriptome analysis of water buffalo *Bubalus bubalis*, an important player in the agriculture, dairy and meat industries in the Indian sub-continent.

Minisatellite 33.15 (5' CACCTCTCCACCTGCC 3') originating from the human myoglobin gene (7q35-q36) has been studied in a number of species [16-18]. This repeat has also been found to be associated with the heterochromatic sequences of the human Y chromosome [19]. In our previous *in-silico *study, we demonstrated presence of minisatellite 33.15 within the transcriptomes of several eukaryotes. Following this, we isolated and characterized several known and novel mRNA transcripts tagged with the consensus of 33.15 repeat loci from different somatic tissues and gonads of water buffalo *Bubalus bubalis *[20].

Owing to the envisaged participation of the spermatozoal mRNA transcripts during early zygotic and embryonic development, we studied the spermatozoal transcriptome of water buffalo *Bubalus bubalis *tagged with minisatellite 33.15 employing Minisatellite associated sequence amplification (MASA). These mRNA transcripts were further characterized for their sequence organization, homology status, variation of gene expression, copy number and evolutionary status of the corresponding genes.

## Results

### Differential distribution of the consensus sequence of 33.15 repeat loci within the spermatozoal and somatic transcriptomes

**Figure 1:**
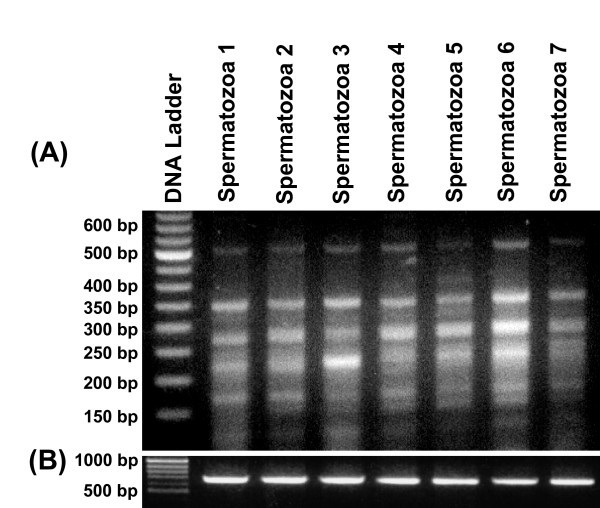
**Minisatellite associated sequence amplification (MASA) performed using oligo based on the consensus of 33.15 repeat loci and cDNA from the spermatozoa of 7 different animals**. Four to seven transcripts in the range of 0.15 kb to 0.5 kb were detected **(A)**. β-actin was used as an internal control with cDNA from all the samples **(B)**.

In previous study, we identified 6 different mRNA transcripts from various tissues of water buffalo using oligos based on the consensus of 33.15 repeat loci [20]. In the present study, we used this oligo (5' CACCTCTCCACCTGCC 3') for MASA and uncovered a total of 72 amplicons comprising 12 different mRNA transcripts from the spermatozoa (Figure 1). Cloning and sequencing followed by the BLAST search of these transcripts showed that 1 had no homology with the sequences in the GenBank and 10 were similar to uncharacterized BAC clones originating from cattle *Bos taurus*, human *Homo sapiens *and mouse *Mus musculus*. Only one mRNA transcript was found to be homologous to the Bovine steroid 21-hydroxylase (P-450-c21) gene (Table 1). Interestingly, comparative analysis of these spermatozoal mRNA transcripts showed that none was common to those identified from different somatic tissues, testis and ovary in earlier study [20]. However, cloning and sequencing of ~20 recombinant clones corresponding to each of these 72 fragments from seven different animals demonstrated their consistent presence in the spermatozoa. In order to ascertain the structural, functional and regulatory status of the MASA uncovered genes/gene fragments, we conducted a comprehensive database search for each mRNA transcript independently. The details of these homologous genes, their accession numbers with chromosomal locations (if available), species, position of our cDNA sequences and their possible functions are given in the table 1.

**Table 1:** Detailed analysis of the spermatozoal mRNA transcripts tagged with consensus of 33.15 repeat loci in buffalo *Bubalus bubalis *and their homology or similarity with genes across the species#

**Transcript ID**	**Accession no.**	**Size (In bp)**	**Clone ID**	**Description of Blast search**	**Accession no. of similar genes**	**Gene length (bp)**	**Chromo-Somal location**	**Nucleotide position of 33.6 tagged transcripts**	**Score**	**QC (%)**	**E-value**	**Similarity (%)**
JS1	EU348479	276	pJSC39	1. *Bos taurus *chromosome 1 reference assembly (based on Btau 4.0); featuring in this part similar to uncharacterized protein C3orf44	NC007299.3	161106243	1	119054612–119054872	446	94	5e-112	95
				2. *Homo sapiens *cDNA FLJ30354 fis, clone BRACE2007682	AK054916	1698	---	590–699	77	39	4e-111	76
				3. *Homo sapiens *chromosome 19 clone CTD-2292J18	AC090427	109233	19	72209–72318	77	39	4e-111	76
				4. *Homo sapiens *tetratricopeptide repeat protein 12 (TTC12) gene, alternatively spliced	EF445041	64712	---	10050–10141	55	35	1e-04	74

JS2	EU348480	276	pJSC40	1. *Bos taurus *chromosome 1 reference assembly (based on Btau 4.0)	NC007324.3	53376418	23	277117728–277117989	412	97	3e-113	94
				2. Bovine steroid 21-hydroxylase gene (P-450-c21) gene	M11267.1	6601	23	6323–6583	414	97	9e-113	94
				3. *Ovis aries *cytochrome P-450 steroid 21 hydroxylase (P-450-c21) gene	EF382834	3454	20	3009–3272	411	97	1e-111	94
				4. Sheep cytochrome protein (P450C21C) mRNA	M92836	2020	---	1862–2018	263	57	3e-67	96
				5. Bovine microsomal cytochrome P-450(C21) mRNA	M12918	2157	23	2005–2157	255	55	7e-65	97
				6. *Bos taurus *cytochrome P450, subfamily XXI (CYP21A2), mRNA	NM_174639	2125	23	2005–2125	203	43	8e-51	97

JS3	EU348481	270	pJSC41	1. *Homo sapiens *BAC clone RP11-498O22 from 2	AC012506	197303	2	172668–172905	84.2	83	3e-113	69%

JS4	EU348482	267	pJSC42	No homology								

JS5	EU348483	297	pJSC43	1. *Bos taurus *chromosome 5 reference assembly (based on Btau 4.0)	NC_007303	125847759	5	1233822–1234084	422	87	6e-105	92

JS6	EU348484	522	pJSC44	1. *Bos taurus *chromosome 9 reference assembly (based on Btau 4.0)	NC_007307	108145351	9	103122927–103123426	803	98	0.0	94
				2. Human DNA sequence from clone RP1-230L10 on chromosome 6 Contains part of a novel gene	AL137005	103679	6	54567–54689	77	23	8e-111	76

JS7	EU348485	517	pJSC45	1. *Bos taurus *chromosome 29, reference assembly (based on Btau_4.0),	NC_007330	51998940	29	30255363–30255634	448	76	2e-123	97
				2. *Bos taurus *chromosome 16, reference assembly (based on Btau_4.0),	NC_007314	77906053	16	45151977–45152179	326	40	9e-87	97
				3. *Bos taurus *Y Chr NOVECTOR CH240-223B23 (Children's Hospital Oakland Research Institute Bovine BAC Library (male	AC220322	194127	Y	25812–25932 29557–29670 117307–117421	200	23	5e-48	96
				4. Bos taurus molybdenum cofactor synthesis 3 (MOCS3), mRNA	XM_589014	2797	13	2194–2393	193	21	9e-46	95
				5. *Bos taurus *T cell receptor gamma cluster 2 (TCRG2) gene	AY644518	188109	---	145614–145734 77004-77124 24056-24175 130049–130169	200	23	3e-50	96
				6. *Bos taurus *clone p68.3 MHC class I antigen gene, 5' UTR and partial cds	AF396777	1169	---	548–668	195	23	2e-46	95

JS8	EU348486	537	pJSC46	1. *Bos taurus *chromosome 2, reference assembly (based on Btau_4.0)	NC_007300	140800416	2	34260844–34261131	484	98	2e-134	97
				2. *Bos taurus *chromosome 11, reference assembly	NC007309	110171769	11	--	201	99	3e-49	87
				2. *Bos taurus *chromosome X, reference assembly	NC_007331	88516663	X	51716993–51717467	235	99	1e-59	71
				3. *Bos taurus *ER degradation enhancer, mannosidase alpha-like 2 (EDEM2), mRNA	NM_001101085	3652	13	2472–2652	134	33	2e-30	77
				4. *Bos taurus *similar to mutant UDP-N-acetylglucosamine 2-epimerase/N-acetylman-nosamine kinase (GNE), mRNA	XM_001250216	6177	8	4822–5284	114	80	2e-24	67
				5. *Rhinolophus ferrumequinum *clone VMRC7-44G7	AC149031	192138	---	44739–45277	341	91	8e-49	77
				6.*Felis catus *clone RP86-177C24	AC087421	14'1881	---	4255–4688	199	86	1e-47	70
				7. *Equus caballus *subclone 355H03 3.3 MHC class I antigen gene	DQ083409	13464	ECA20	2473–3016	187	91	6e-44	68

JS9	EU348487	387	pJSC47	1. *Bos taurus *chromosome 10, reference assembly (based on Btau_4.0)	NC_007308	106383598	10	53535034–53535410	616	98	3e-174	97
				2. *Homo sapiens *chromosome 15 clone RP11-358M11 map 15q21.3	AC016525	256019	15q21.3	119191–119420	158	59	2e-35	75

JS10	EU348488	398	pJSC48	1. *Bos taurus *chromosome 2, reference assembly (based on Btau_4.0)	NC_007300	140800416	2	89906829–89907043 38557472–38557648	327	98	2e-87	94 95
				2. *Bos taurus *Y chr BAC CH240-96I24 (Children's Hospital Oakland Research Institute Bovine BAC Library (male)	AC213711	186273	---	149863–150033 117725–117896 29045–29213	282	52	4e-75	96 94 91
				3. *Bos taurus *Fanconi anemia, complementation group M, mRNA	BC149286	1448	---	1228–1398	282	42	2e-75	96
				4. Bos taurus similar to zinc finger protein 568 (ZNF793), mRNA	XM_001254432	5407	18	4744–4914	264	42	5e-70	94
				5. Bos taurus similar to peroxisomal long-chain acyl-coA thioesterase (LOC516537), mRNA	XM_594692	2022	10	1603–1773	264	42	5e-70	94

JS11	EU348489	370	pJSC49	1. Bos taurus chromosome 12, reference assembly (based on Btau_4.0	NC_007310	85358539	12	82142726–82143101	513	98	2e-143	90
				2. *Bos taurus *BAC CH240-10G15 (Children's Hospital Oakland Research Institute Bovine BAC Library (male)	AC149774	186916	---	129128–129350	240	60	2e-60	88
				3. *Bos taurus *cDNA clone IMAGE:8284348 & CR4-NOT transcription complex, subunit 8	BC119978	2730	---	545–742	235	53	5e-61	86
				4. *O. aries *KII-9 gene for hair type II keratin intermediate filament	X62509	7371	---	1823–2030	221	56	2e-54	83
				5. *Bos taurus *similar to Lysophosphatidic acid receptor Edg-7 (LPA receptor 3) (LPA-3) (EDG7), mRNA	XM_612024	2913	3	2138–2356	219	58	7e-54	82
				6. *Bos taurus *similar to Myotubularin-related protein 2, mRNA	BC148076	5057	---	4447–4665	215	58	9e-53	82

JS12	EU348490	375	pJSC50	1. *Bos taurus *chromosome 16, reference assembly (based on Btau_4.0)	NC_007314	77906053	16	1730212–1730571	563	96	2e-158	94

### Conserved 33.15 tagged spermatozoal genes are present as single to double copy in buffalo genome

**Figure 2:**
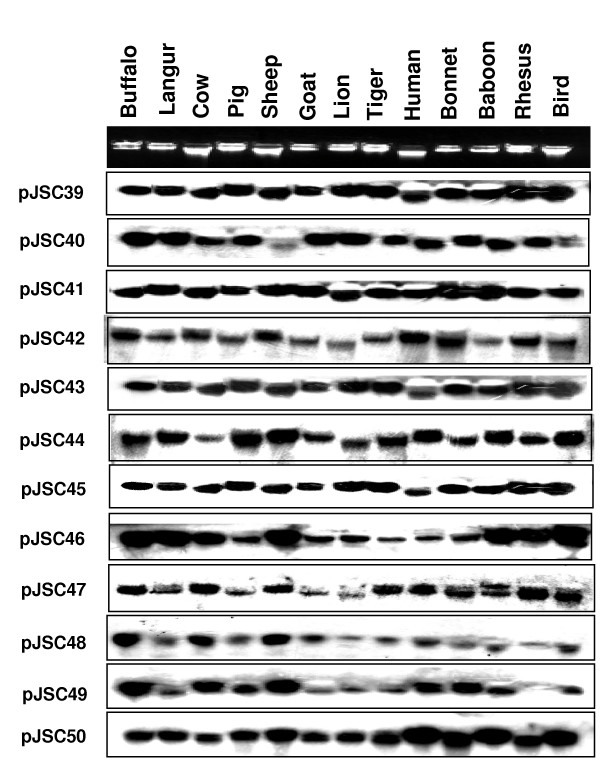
**Cross-hybridization of genomic DNA from different species with the recombinant clones corresponding to different gene fragments uncovered by MASA**. The names of the species are given on top of the panel and recombinant clones corresponding to genes/gene fragments, on the left.

To determine possible evolutionary significance of MASA uncovered genes/gene fragments, we conducted cross-hybridization studies using individual recombinant clone containing corresponding gene fragment with the gDNA of 13 different species (Figure 2). All the 33.15 tagged spermatozoal genes were found to cross hybridize with all the thirteen species suggesting their ubiquitous roles in the higher eukaryotes.

Following sequence analyses and conservation studies, the copy number status was calculated for these genes/gene fragments using standard curve method and SYBR green chemistry. Extrapolation of the standard curves obtained with 10 fold dilution series of the respective recombinant plasmids containing corresponding gene fragments (Additional file 1A–B) demonstrated their 1 to 2 copies per haploid genome in buffalo (Table 2).

**Table 2:** Relative quantitative expression and copy number status of the spermatozoal genes/gene fragments tagged with the consensus of 33.15 repeat loci

**S. No.**	**Trans. ID**	**Gene Accession**	**Relative Expression in Different Tissues and Spermatozoa (In folds)**													**Copy Number**
				
		**Numbers**	**Testis**	**Ovary**	**Spleen**	**Liver**	**Lung**	**Kidney**	**Heart**	**Forebrain**	**Hindbrain**	**Spermatozoa of Four Different Animals**				**Status Per Haploid**
												**SP1**	**SP2**	**SP3**	**SP4**	**Genome**
1.	JS6	EU348484	181	34	6	14	21	12	1	10	79	1260	1552	2195	1910	**1–2 copies**
	
2.	JS7	EU348485	45	5	5	16	8	3	1	3	18	477	675	349	675	
	
3.	JS8	EU348486	388	181	68	337	388	157	1	78	326	10809	4096	12417	12417	
	
4.	JS12	EU348490	24	1	5	10	1	14	2	4	5	256	238	831	955	
	
5.	JS9	EU348487	194	1	3	6	1	21	12	3	3	1024	4096	1176	4096	
	
6.	JS11	EU348489	111	9	6	10	1	10	15	7	6	2352	7131	6655	1351	
	
7.	JS10	EU348488	97	1	1	3	2	20	*2*	3	4	2048	6654	4388	5095	
	
8.	JS5	EU348483	137	2	1	5	1	15	8	15	15	955	831	2095	832	
	
9.	JS4	EU348482	2195	158	104	74	52	45	1	5	152	17560	8192	12884	19616	
	
10.	JS3	EU348481	128	16	3	2	1	16	2	24	22	1448	1448	630	1902	
	
11.	JS2	EU348480	29	3	28	17	1	*9*	26	48	59	831	776	315	477	
	
12.	JS1	EU348479	181	30	2	3	1	*10*	6	25	30	3821	2896	2702	3042	

### Differential expression profiles of 33.15 tagged mRNA transcripts

**Figure 3:**
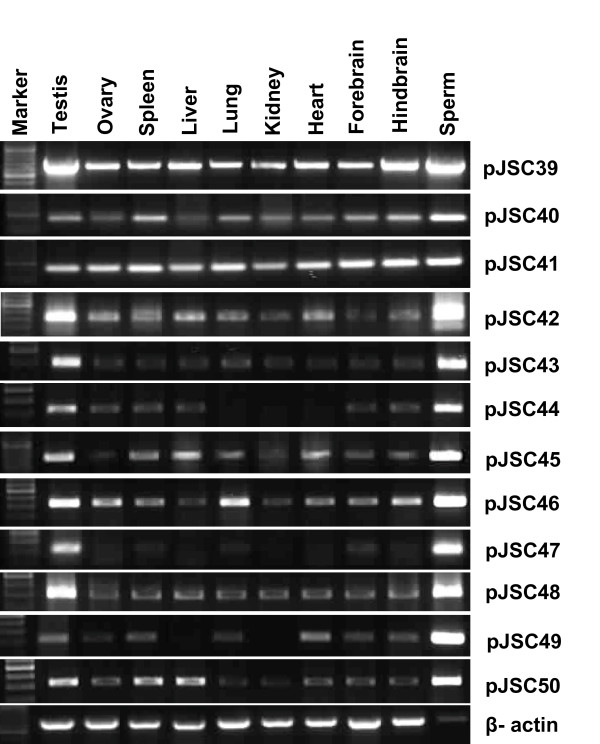
**RT-PCR analyses for the 33.15 tagged mRNA transcripts using internal primers and cDNA from different somatic tissues, gonads and spermatozoa**. Transcript IDs are given on the right and names of the tissues on top. Quality and quantity of the cDNA samples were normalized by PCR with β-actin derived primers. Tissue specificities of the transcripts were ascertained on the basis of presence or absence of the amplicons using corresponding cDNA templates which were further confirmed by Real Time PCR and Southern blotting.

Comparative expression profiles of the uncovered genes were studied to explore their functional status in different somatic tissues, gonads and spermatozoa. The expression study for each MASA uncovered gene/gene fragments was done first by RNA slot blot hybridization (Not shown) and later by RT-PCR analysis (Figure 3). The quantitative expressional analysis was then performed for individual transcript using equal amount of cDNA templates and *GAPDH *as an internal control employing SYBR green assays in Real Time PCR.

The primer specificity for each individual gene was established using five fold dilution series of the template cDNA (Additional file 1C–D). Straight standard curve with a slope = (-3.4) to (-3.6) and a single peak in the dissociation protocol established the maximum specificity and efficiency of the primers. For more accuracy, the expression study was conducted using three different dilutions of cDNA. The relative quantitation of expression so obtained was substantiated further using somatic/gonadal/spermatozoal cDNA from five additional animals.

**Figure 4:**
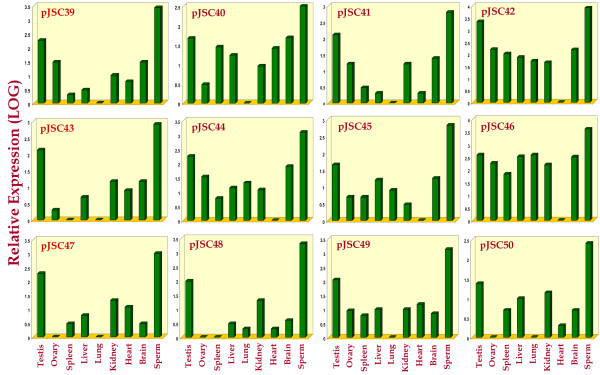
**Comparative expression of 33.15 tagged genes/gene fragments**. Bars demonstrating expression levels across the tissues and spermatozoa of buffaloes represented in different colors corresponding to different mRNA transcripts. Note the highest expression of most of the fragments in the testis and/or spermatozoa.

The relative expression study amongst all the somatic tissues, gonads and spermatozoa demonstrated the highest and/or unique expression of all the 12 transcripts in the spermatozoa including that of pJSC40 [Coding for buffalo steroid 21-hydroxylase (P-450-c21) gene, GenBank accession number: EU348480]. When expression was compared amongst different somatic and gonadal tissues, only one transcript showed uniform expression and the other one, highest expression in testis, liver, lung & brain. The remaining ten transcripts showed highest expression in the testis compared to that in other somatic tissues and ovary (Figure 4 and Table 2). Significantly, 4 (pJSC42, pJSC44, pJSC45 and pJSC46) of the 12 uncovered genes showed negligible expression in the forebrain but comparatively higher expression (18 to 326 folds) in the hindbrain of buffalo.

## Discussion

Minisatellites have been implicated with gene regulation, chromosomal fragile sites and genome imprinting [15,21,22] but the biological significance of their association with the coding regions remains largely unresolved. In the present study, we established the association of 33.15 repeats with the spermatozoal transcriptome of water buffalo, *Bubalus bubalis *and observed highest expression of these transcripts in testis and/or spermatozoa indicating their crucial roles in male gametogenesis.

### Potential implications of association of repeats with the spermatozoal transcriptome

Our previous study showed distribution of 33.15 sequences in the flanking regions and within the mRNA transcripts of many structural, functional and regulatory genes in buffalo and six other species [20]. Previously, various 33.15 tagged mRNA transcripts were uncovered from somatic tissues and gonads of buffalo. Here, a total of 12 different mRNA transcripts, representing one known and others mostly uncharacterized ones from spermatozoa were identified (Figure 1 and Table 1) complementing our earlier study. Notably, none of the transcripts was shared between somatic/gonadal tissues and spermatozoa. The differential transcript profile may be a reflection of their diverse functions in somatic tissues, gonads (testis/ovary), and spermatozoa. Alternatively, they may have specific roles to play during different stages of development. These transcripts could not be picked up in somatic tissues/gonad and vice-versa due to either polymorphic nature of SSRs or their much lower numbers. A comparison of the mRNA transcripts uncovered in the present study with those reported earlier [3] confirmed that these were the different ones.

Thus, present study can be used as a basis for evaluation of other repeats to unveil their combined significance within and adjacent to the coding regions. Spermatozoa have come a long way from being regarded as an artifact to a repository of a variety of RNAs. The initial controversy which shrouded the existence of spermatozoal RNA pool has now been cleared by the identification of several transcripts in different species including human [2-7].

Many signaling molecules and transcription factors have been identified which are transported into the zygotic cytoplasm upon fertilization by the spermatozoa [2-7]. However, several other spermatozoal transcripts remain to be characterized. Present study seems to be the first one, unveiling existence of the minisatellite tagged transcripts in the buffalo spermatozoa suggesting their predominant involvements in the pre- and post-fertilization events. It becomes more significant, when these transcripts showed faithful conservation across thirteen different species suggesting their testicular functions in much broader number of higher eukaryotes.

### Prospects of repeat tagged transcripts carrying highest expression in the testis and spermatozoa

All the 33.15 tagged genes/gene fragments showing highest expression in the testis and spermatozoa suggested their strong participations in the testicular development and spermatogenesis. Further, uniform expression of steroid 21-hydroxylase gene suggested its consistent requirement in all the tissues except spermatozoa. The steroid 21-hydroxylase; P450 (C21) belongs to the cytochrome P-450 super-gene family and plays a crucial role in the synthesis of steroid hormones such as cortisol and aldosterone. Mutations in this gene have been implicated in causing Adrenal Hyperplasia [1].

This gene has been reported to show maximum expression in adrenal glands and minimum in liver and testis. However, in our study, this gene showed maximum expression in spermatozoa thus, adding a new dimension towards its unidentified functions in the sperm development and maintenance. Expression of P450 (C21) gene could not be confirmed in the adrenal gland of buffalo due to unavailability of the tissue sample. Since the spermatozoal mRNA is delivered to the ooplasm, and has its potential roles in the chromatin repackaging, early zygotic and embryonic development and post syngamy events [2,3,5,23], the observed highest expression of all the 33.15 tagged mRNA transcripts in spermatozoa suggest their important roles towards specific functions of paternal genome sustenance.

## Conclusion

Present work demonstrated the association of consensus sequence of minisatellite 33.15 with buffalo spermatozoal transcriptome. Maximum expression of all the 33.15-tagged mRNA transcripts in testis and spermatozoa suggested their involvement in various testicular functions and fertilization. Following this approach, other repeat tagged mRNA transcripts may be studied within the buffalo genome and across the species. A comparative mRNA fingerprint based on several minisatellites resulting in detailed expression profile would allow the identification of common and uncommon transcripts in somatic tissues and gonads. Indeed, this would help understanding the more focused roles of repetitive DNA sequences in the paternal genome during and post fertilization events and different stages of embryonic development.

## Methods

### Sperm purification and RNA isolation

Fresh ejaculated semen samples of buffaloes were obtained from the local dairy farm following strictly the guidelines of the Institute's Bio-Ethical and Biosafety Committees. All the sperm samples were procured from the buffalo *in-vitro *fertilization (IVF) center where utmost care was taken to avoid the diploid cell contaminations in the sperm preparations using highly sensitive methodologies and modern techniques. Samples were subjected to percoll gradient method to select only motile sperms as described earlier [23]. Sperm cells were washed in sperm wash buffer (0.15 mM NaCl and 10 mM EDTA) and pellets were processed for RNA extraction using TRIzol reagent (Invitrogen, CA). After re-suspension of the cells in TRIzol reagent, cells were incubated at 60°C for 30 min as described earlier [23], vortexing every 10 min for lysis following manufacturer's instructions. Each RNA sample was treated with RNase free DNase I (Ambion, USA), and precipitated with ammonium acetate and ethanol following standard protocols [23-25].

Quantification of RNA was done with spectrophotometer (Amersham Life Sciences). A fraction of each RNA sample (5 ng/μl) was tested for residual DNA contamination by PCR using primers against β-actin (*ACTB*; GenBank accession no. DQ661647) and Protamine-1 (*PRM1*; GenBank accession no. NM_174156) following standard procedures [23,25] (Additional file 2). These exon-specific primers encompassed one intron, thus, distinguishing cDNA from the genomic sequence. The PCR was conducted using initial denaturation at 95°C for 5 min; 35 cycles of denaturation at 94°C for 30 sec, primer annealing at 55°C for *PRM1 *and 60°C for *ACTB *for 30 sec, extension at 72°C for 1 min; and final extension at 72°C for 10 min.

### Isolation of genomic DNA, total RNA and cDNA synthesis

Blood and tissue samples of both the sexes of water buffalo were collected from local slaughterhouse, following guidelines of the Institute's Ethical and Bio-safety Committees. Details of the genomic DNA isolation from buffalo and other species used in this study for cross hybridization have been reported earlier [20,26]. Total RNA was also isolated from Testis, Ovary, Spleen, Liver, Lung, Kidney, Heart and Brain using TRIzol reagent (Invitrogen, CA) as per the standard procedure [23-25]. Following quantification of RNA, the cDNA synthesis was conducted using commercially available cDNA archive kit (ABI, USA). The quality of cDNA so prepared was confirmed by PCR amplification using β-actin and *PRM1 *primers. The absence of somatic and other non-sperm cells in each sperm RNA preparation was confirmed by PCR reactions using each cDNA sample, and common leukocyte antigen (*CD45*) and epithelial E-cadherin (*CDH1*) gene markers (Additional files 2 &3) at 55°C annealing temperature.

### Minisatellite associated sequence amplification (MASA)

For conducting minisatellite associated sequence amplification (MASA), a 16 base long oligo (5' CACCTCTCCACCTGCC 3') based on consensus of 33.15 repeat loci, was purchased from Microsynth GmbH (Balgach, Switzerland). MASA reactions were performed using cDNA templates from the spermatozoa of different animals following standard procedures [20,26]. The resultant amplicons were resolved on 20 cm long 2.5% (w/v) agarose gel using 0.5× TBE buffer.

### Cloning, sequencing and characterization of MASA uncovered amplicons

From the MASA reactions, a total of 72 amplicons were uncovered (Table 1). These amplicons resolved on the agarose gel were sliced; DNA eluted (Qiagen Gel Extraction kit, Germany) and processed independently for cloning into pGEMT-easy vector (Promega, USA). A total of 20–25 recombinant clones corresponding to each amplicon were characterized by restriction digestion and slot blot hybridization using labeled buffalo genomic DNA [27]. Sequences of all the clones generated from every single amplicon were independently subjected to ClustalW alignment to ascertain interclonal variation. Finally, a total of 12 sequences were deposited in the NCBI GenBank and their accession numbers are given in the table 1.

Database search was conducted to determine homology of these sequences independently with other entries in the GenBank using default NCBI server  having megablast "highly similar" and blastn "somewhat similar" algorithms. Each sequence was first subjected to blast search across the databases of Nucleotide collection (nr/nt), Reference mRNA sequences (refseq_mRNA), NCBI genomes (chromosome) and Expressed sequence tags (ESTs) of all the organisms, and thereafter individually to those of cattle, sheep, goat and human.

### Cross-hybridization of the uncovered genes/gene fragments

Cross hybridization studies were conducted using DNA from across the species. DNA was extracted from the peripheral blood of buffalo *Bubalus bubalis*, cattle *Bos indicus*, sheep *Ovis aries*, goat *Capra hircus*, human *Homo sapiens*, Pigeon *Columba livia*, pig *Sus scrofa*, Baboon *Papio hamadryas*, Bonnet monkey *Macaca radiata*, Langur *Presbytis entellus*, Rhesus monkey *Macaca mulatta*, Lion *Panthera leo*, Tiger *Tigris tigris *following standard protocols [25,26]. Lion and Tiger blood samples were procured with due approval of the competent authorities of the States and Union Government of India. Hybridization of genomic DNA from different sources using recombinant cloned probes was conducted following standard procedures [25,27].

### RNA slot blot analysis, RT-PCR and Southern Blotting

For RNA slot blot analysis, approximately 2 μg of total RNA from different tissues of buffalo in 100 μl of 2 × SSC was slot blotted onto the nylon membrane (Minifold Apparatus, Schleicher & Schuell, Germany) and UV fixed. For positive control, 5 ng of recombinant plasmid, each, was included in the blot(s). Hybridizations were performed under high stringent conditions using standard procedure [25,26]. Individual probes for each fragment was labeled with [^32^P] α-dCTP using rediprime™ II kit (Amersham Pharmacia Biotech, USA). In order to confirm the RNA slot blot, internal primers were designed from each fragment (Additional file 4) and RT-PCR was conducted using cDNA templates from different tissues following standard thermal profiles (Additional file 4). The products were resolved on the agarose gel and transferred onto the nylon membrane followed by hybridization with [^32^P] α-dCTP labeled recombinant clones corresponding to each uncovered fragment using standard procedures [25-27]. Bubaline derived β-actin gene probe and bacterial genomic DNA were used as positive and negative controls, respectively.

### Relative Expression Studies Using Real Time PCR

For expression quantification of different genes/fragments (JS1–JS12), SYBR green assays were conducted using Real Time PCR (Sequence Detection System, 7500, ABI) for individual fragments using equal amount of cDNA from all the tissues and spermatozoa. The *GAPDH *and *ACTB *genes were used as endogenous controls for relative expression. The primers for assessing relative expression of each of the transcripts (JS1–JS12, *GAPDH *&*ACTB*) and the copy numbers of their corresponding genes in the haploid genome were designed using Primer Express 3.0 software (Applied Biosystems, USA). The primer details have been given in additional file 4. Standard curves for all the genes were composed of five points covering 10 log orders of magnitude using a serial dilution of a purified cDNA template. Slopes of these standard curves were obtained between -3.3 and -3.6, reflecting amplification kinetics ranging from 80% to 100%.

Melting-curve analysis was performed for SYBR Green amplifications by plotting fluorescence intensity in a graphic model. To validate the specificity of the qRT-PCR assays, a single melting temperature peak representing a single amplicon (SYBR Green amplifications) was confirmed. Thereafter, the quantitative RT-PCR assay was performed individually for JS1–JS12, *GAPDH *and *ACTB *genes (Additional file 4) using SYBR Green chemistry containing 5 μl of 2× Power SYBR Green Master Mix (Applied Biosystems), 0.3 μM of forward and reverse primers each, and 0.25 U of AmpErase UNG (Applied Biosystems). All the runs included a triplicate of the melting curve for each sample and from three to six negative controls (absence of target DNA) that were partitioned to detect potential contamination during preparation of the plate. Thermal cycling conditions used were: 50°C for 2 min (incubation for the AmpErase UNG) and then the first denaturation step at 95°C for 10 min, followed by 40 cycles of 95°C for 15 sec and 60°C for 1 min. Further, each experiment was repeated three times at different concentrations of the template to ensure consistency of the results. Expression level of the genes was calculated using the formula: expression status = (1+E)^-ΔΔCt^, where E is the efficiency of the PCR and ΔΔCt is the cycle threshold normalized first with the endogenous control *GAPDH *(Ct sample - Ct GAPDH = ΔCt) and then with the calibrator sample (ΔCt Sample - ΔCt Calibrator = ΔΔCt) [23,25,26].

### Copy number calculation of the 33.15 tagged genes using Real Time PCR

The copy number status of the genes/gene fragments corresponding to each 33.15 tagged transcripts was calculated based on absolute quantification assay using SYBR green chemistry. The primers standardized and checked for the quantitative expression for JS1–JS12 were employed also for copy number calculation (Additional file 4). The gene copy number of individual genes/gene fragments corresponding to the transcripts tagged with minisatellite 33.15 was calculated using standard curve method. A ten fold dilution series of known copies of each recombinant clone (for individual JS1–JS12) was used to plot standard curve with slope -3.3 to -3.4 substantiating maximum efficiency of the real time PCR reaction. Extrapolation of standard curves for individual recombinant clones uncovered copy number status of each 33.15 tagged gene in the haploid buffalo genome.

## Authors' contributions

JS performed the experiments and in-silico analysis, analyzed and interpreted the data, and wrote the manuscript. SP performed the experiments and in-silico analysis, analyzed and interpreted the data. SK Provided the research samples for performing the experiments and intellectual inputs on the manuscript. SA designed the concept, scrutinized the data analysis, finalized the manuscript and figures and provided overall supervision. All authors have read and approved the final manuscript

## Supplementary Material

Additional file 1 **Representative Real Time PCR amplification plots for copy number calculation using target DNA and 10 fold dilution series (From 300 million to 300 copies) of recombinant plasmids containing genes tagged with 33.15 sequence**. **(A)**. The assays were performed using SYBR green chemistry and the derived Standard **(B)**. Dissociation or Melting **(C) **and Standard Curves **(D) **were deduced for quantification of expression using five fold dilution series of the cDNA samples. Single peak in the dissociation curve conforms to the high specificity of the primers. Click here for file

Additional file 2 **List of primers used for the verification of any genomic or RNA contamination in sample**. The primers for *ACTB *were designed by us and for *CD45 *and *CDH1 *genes, primers were based on earlier report (23). These primers also spanned several introns but their positions were not defined. Click here for file

Additional file 3 **PCR analysis verifying the absence of contamination**. The contamination of possible gDNA in the RNA and cDNA was checked using *ACTB *&*PRM1 *markers. Likewise, CD45 and CDH1 markers were used to check contamination of any somatic cells in the spermatozoal RNA and cDNA of all the animals. The RNA and cDNA of respective animal, for e.g. SP1 R represents RNA of spermatozoa of first animal and SP1 C for cDNA of the same animal, has been given in figure. Click here for file

Additional file 4 **Details of the oligos used for RT-PCR, Copy number Calculation and Relative Expressional Studies**. The respective sequences (5'-3') of forward and reverse primers and their annealing temperatures are also given. Click here for file
