# Supplementary figures and images for: Expressional dynamics of minisatellite 33.15 tagged spermatozoal transcriptome in Bubalus bubalis
Source: BMC Genomics. 2009 Jul 7;10:303. doi: 10.1186/1471-2164-10-303 (PMC2713999; doi:10.1186/1471-2164-10-303)

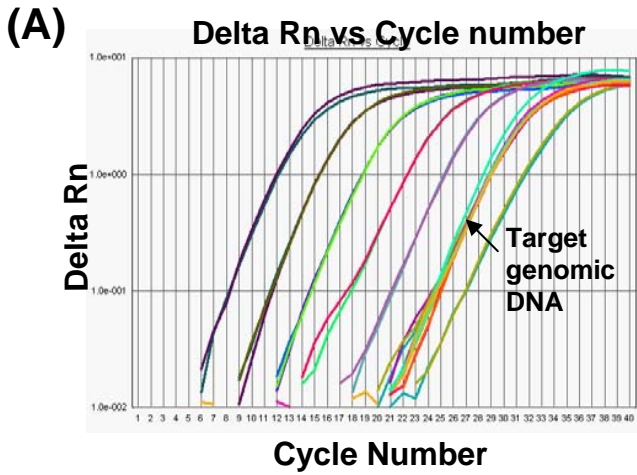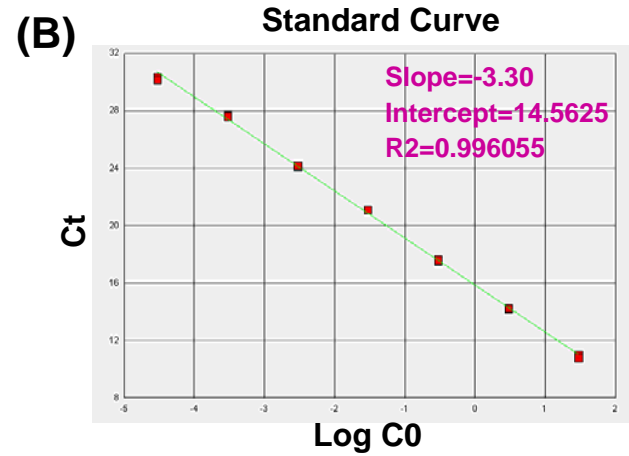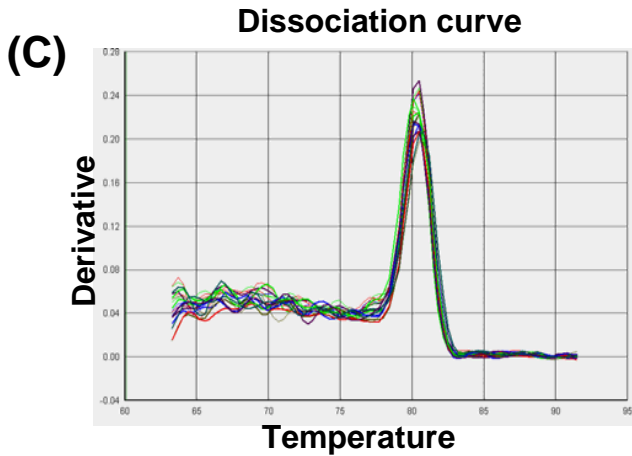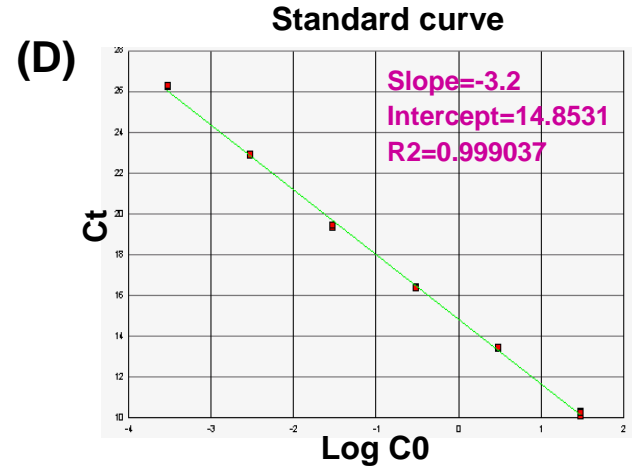

**Additional file 1**

Supplement: Additional file 1 — Representative Real Time PCR amplification plots for copy number calculation using target DNA and 10 fold dilution series (From 300 million to 300 copies) of recombinant plasmids containing genes tagged with 33.15 sequence. (A). The assays were performed using SYBR green chemistry and the derived Standard (B). Dissociation or Melting (C) and Standard Curves (D) were deduced for quantification of expression using five fold dilution series of the cDNA samples. Single peak in the dissociation curve conforms to the high specificity of the primers. [file 1471-2164-10-303-S1.pdf]

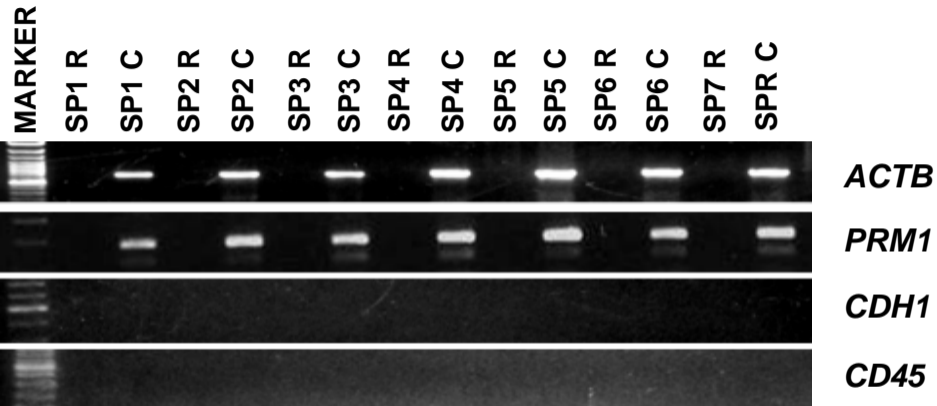

**Additional file 3**

Supplement: Additional file 3 — PCR analysis verifying the absence of contamination. The contamination of possible gDNA in the RNA and cDNA was checked using ACTB &PRM1 markers. Likewise, CD45 and CDH1 markers were used to check contamination of any somatic cells in the spermatozoal RNA and cDNA of all the animals. The RNA and cDNA of respective animal, for e.g. SP1 R represents RNA of spermatozoa of first animal and SP1 C for cDNA of the same animal, has been given in figure. [file 1471-2164-10-303-S3.pdf]
