# Supplementary material for: Expressional dynamics of minisatellite 33.15 tagged spermatozoal transcriptome in Bubalus bubalis
Source: BMC Genomics. 2009 Jul 7;10:303. doi: 10.1186/1471-2164-10-303 (PMC2713999; doi:10.1186/1471-2164-10-303)
Supplement: Additional file 2 — List of primers used for the verification of any genomic or RNA contamination in sample. The primers for ACTB were designed by us and for CD45 and CDH1 genes, primers were based on earlier report (23). These primers also spanned several introns but their positions were not defined. [file 1471-2164-10-303-S2.pdf]

**Additional File 2: List of primers used for the verification of any genomic or RNA contamination in sample.**

| Gene | Accession numbers | Primer Sequence (5'-3') | Product size (bp) | |
|---|---|---|---|---|
| | | | cDNA | gDNA |
| *ACTB* | DQ661647 | Forward 5'CAGATCATGTTCGAGACCTTCAA3'<br>Reverse 5'GATGATCTTGATCTTCATTGTGCTG3' | 630 | 720 |
| *PRM1* | NM_174156 | Forward 5'AGATACCGATGCTGCCTCAC3'<br>Reverse 5'GTGGCATGTTCAAGATGTGG3' | 234 | 334 |
| *CD45* | AJ400864 | Forward GACATCGCAGTGTTTGTTGC<br>Reverse GGAGGTTCACATTCCTCTCG | 229 | Undefined |
| *CDH1* | NM_001002763 | Forward TCTACAGCATCACTGGCCAACGAGCTG<br>Reverse TGCTTGGACCATCAGGGTGTATGTGGG | 476 | Undefined |

*The primers for *ACTB* were designed by us, however, for *CD45* and *CDH1* genes were considered from Lalancette *et al*. These primers also spanned several introns but their positions were not defined.
