# Supplementary material for: Expressional dynamics of minisatellite 33.15 tagged spermatozoal transcriptome in Bubalus bubalis
Source: BMC Genomics. 2009 Jul 7;10:303. doi: 10.1186/1471-2164-10-303 (PMC2713999; doi:10.1186/1471-2164-10-303)
Supplement: Additional file 4 — Details of the oligos used for RT-PCR, Copy number Calculation and Relative Expressional Studies. The respective sequences (5'-3') of forward and reverse primers and their annealing temperatures are also given. [file 1471-2164-10-303-S4.pdf]

Additional file 4: Details of the Oligos used for RT-PCR, Copy number Calculation and Relative Expressional Studies

S.N.	Oligo ID	Gene ID	Sequence (5'-3')	Annealing Temp. (in ^{O}C)
**A. Internal Primers Designed from the Identified Genes/Gene Fragments for RT-PCR studies**				
1.	JSO1485	pJSC39	F 5' CTTAACCCGGCTTCAGCATCT 3'	59
2.	JSO1486		R 5'ACTCCTAGAAGTTGGTTCTGGTC3'	
3.	JSO1487	pJSC40	F 5'TGGAACAATAATGCCAATCTCAC3'	61
4.	JSO1488		R 5'GTTCCAGAAATCAATCAGCCATT3'	
5.	JSO1489	pJSC41	F 5'ACTTCACAGAGCTGATGGAAACA3'	60
6.	JSO1490		R 5'AGACGTCTCAATCCACATTCG3'	
7.	JSO1491	pJSC42	F 5'ACCTGAACCTGCTGTTTGAATTT3'	61
8.	JSO1492		R 5'ACACCAAGACTTTCTGCCTCTCT3'	
9.	JSO1493	pJSC43	F 5'CACGTTCAGTTAAACATTTGTGGA3'	61
10.	JSO1494		R 5'CATATCCAACCTTCTTTCTGTCCT3'	
11.	JSO1495	pJSC44	F 5'TGTTTTCTCATTCACTCAGGTTCA3'	61
12.	JSO1496		R 5'ACATGTGGCCTTTGAATACTTCTC3'	
13.	JSO1497	pJSC45	F 5'CCAAAGTACTGGAGTTTCAGCTTC3'	60
14.	JSO1498		R 5'ATCCTAGCTCTGCTCATCTCATGT3'	
15.	JSO1499	pJSC46	F 5'GAAGTTGTGGAGAAAAAGGGAACTT3'	61
16.	JSO1500		R 5'TAATGCCTTCAAGGTTCATCCATA3'	
17.	JSO1501	pJSC47	F 5'CTTTCTTCTTCCTCATTCACACCT3'	60
18.	JSO1502		R 5'ATCTTTCCAAGTCTGTATCCACCA3'	
19.	JSO1503	pJSC48	F 5'TGTCTGTGTTTTGCTTTTGAGAAA3'	59.5
20.	JSO1504		R 5'GCATCAGAGTCTTTTCCAATGAGT3'	
21.	JSO1505	pJSC49	F 5'ATGCAGAAACAGAGGAAAACAGTC3'	61.5
22.	JSO1506		R 5'TCACATCAGGTGACCAAAATACTG3'	
23.	JSO1507	pJSC50	F 5'AGGGGTGATGGAGGACTTAAAAT3'	60
24.	JSO1508		R 5'CACAAACACACTCAGGGTAGTTCC3'	
25.	JSO1509	*ACTB*	F 5'CAGATCATGTTCGAGACTTCAA3'	60
26.	JSO1510		R 5'GATGATCTTGATCTTCATTGTGCTG3'	
**B. Real Time Primers for Copy Number Calculation and Relative Expressional Studies**				
1.	JSR760	pJSC39	F 5' ACAGGCGTAACCCAGGCTATAA 3'	60
2.	JSR761		R 5' GGGAGGCTTACCTGTTTTTGC 3'	
3.	JSR758	pJSC40	F 5' GACGGCTCAGGATTCTGCTT 3'	60
4.	JSR759		R 5' CTCCCTGTCTCCCCTTTCC 3'	
5.	JSR756	pJSC41	F 5' CAATGCAGCCCGGTAAGG 3'	60
6.	JSR757		R 5' TTAATCCCCTCACCCCATC 3'	
7.	JSR754	pJSC42	F 5' CAGAGAGAGGCAGAAAGTCTTGGT 3'	60
8.	JSR755		R 5' CCATCTTCACTGTGCTGCTAAAA 3'	
9.	JSR752	pJSC43	F 5' ACGTTCAGTTAAACATTTGTGGATCT 3'	60
10.	JSR753		R 5' AGGACCTACTCCATTTTCAATCTTTAGT 3'	
11.	JSR738	pJSC44	F 5' CATGCTGAGCCAGAGGTCAA 3'	60
12.	JSR739		R 5' AAGTCAAGAATCGCACCAAGAGT 3'	
13.	JSR740	pJSC45	F 5' GTGGGATCGGAGCTTATCGA 3'	60

14.	JSR741		R 5’ GGTATCTGGAGTGAAAACAAACA 3’	
15.	JSR742	pJSC46	F 5’ CTCAAAATTAGTTCACTGTAGCATCATTT 3’	60
16.	JSR743		R 5’ CAGATATTCTTTATCCATACACCACCAA 3’	
17.	JSR746	pJSC47	F 5’ AAGGCAGCAATGGTGGATACA 3’	60
18.	JSR747		R 5’ TCTTCAGGCACCCCTCCTAA 3’	
19.	JSR750	pJSC48	F 5’ AGATCAGCCCTGGGATTTCTTT 3’	60
20.	JSR751		R 5’ CGCATGAGGTGGCCAAA 3’	
21.	JSR748	pJSC49	F 5’ AGGCCCTTGGACAGCAAAG 3’	60
22.	JSR749		R 5’ TCATAGCATCAGTCATCCCAATAATTA 3’	
23.	JSR744	pJSC50	F 5’ CTTTCTTGGTGTGTCCATGTTGA 3’	60
24.	JSR745		R 5’ GCCACAAACACACTCAGGGTAGT 3’	
25.	JSR1386	GAPDH	F 5’ GCAAGTTCCACGGCACAGT 3’	60
26.	JSR1387		R 5’ GATGGTGATGGCCTTTCCAT 3’	
